# Supplementary material for: Modelling the supply and need for health professionals for primary health care in Ghana: Implications for health professions education and employment planning
Source: PLoS One. 2021 Sep 28;16(9):e0257957. doi: 10.1371/journal.pone.0257957 (PMC8478216; doi:10.1371/journal.pone.0257957)
Supplement: S1 Table — (DOCX) [file pone.0257957.s001.docx]

**Supplementary material 2: Summary table on level of health evidence (prevalence of diseases and risk factors)**

| **No** | **Disease, Risk Factor, Public Health Intervention** | **Type of indicator** | **Value  (Recent Estimate)** | **Lower Bound** | **Upper Bound** | **Year of the recent estimate** | **Value  (Previous estimate)** | **Year of the previous estimate** | **Reference** | **Web-link 1** | **Web-link 2** |
| --- | --- | --- | --- | --- | --- | --- | --- | --- | --- | --- | --- |
|  | Influenza and Pneumonia (general population) | Prevalence | 4.0% | 4.0% | 4.0% | 2016 | 4.0% | 2014 | (GSS et al., 2015: 113) | <https://dhsprogram.com/publications/publication-FR307-DHS-Final-Reports.cfm> |  |
|  | Coronary Heart Disease | Prevalence | 17.0% | 7.2% | 17.0% | 2017 | 7.2% | 2005 | (Appiah et al., 2017) | <https://onlinelibrary.wiley.com/doi/full/10.1002/clc.22753> |  |
|  | Stroke (50+ years) | Prevalence | 4.6% | 2.6% | 4.6% | 2016 | 2.6% | 2007 | (Sanuade et al., 2019) | <https://www.ncbi.nlm.nih.gov/pmc/articles/PMC6415815/> | <http://ugspace.ug.edu.gh/handle/123456789/22927> |
|  | HIV/AIDS (15- 49 years) | Prevalence | 1.7% | 1.7% | 2.0% | 2019 | 1.7% | 2014 | (Ghana AIDS Commission, 2019; GSS et al., 2015) | <https://www.ghanaids.gov.gh/pages/fact-sheets-reports> |  |
|  | Malaria (all ages) | Prevalence | 36.0% | 36.0% | 47.0% | 2014 | 36.0% | 2011 | (GSS et al., 2015) | <https://dhsprogram.com/publications/publication-FR307-DHS-Final-Reports.cfm> |  |
|  | Tuberculosis (All ages) | Prevalence | 1.7% |  |  | 2017 | 1.7% | 2013 | (Addo et al., 2019) | <https://www.scirp.org/journal/paperinformation.aspx?paperid=93384> | <http://ghdx.healthdata.org/record/ghana-national-tuberculosis-prevalence-survey-2013> |
|  | Low Birth Weight (% of live births) | Incidence | 10.0% | 10.0% | 29.6% | 2015 | 10.0% | 2014 | (Abubakari et al., 2015; GSS et al., 2015) | <https://www.ncbi.nlm.nih.gov/pmc/articles/PMC4681076/> |  |
|  | Road Traffic Accidents (% of motorists) | Incidence | 4.0% |  |  | 2011 |  |  | (Konlan et al., 2020; Kudebong et al., 2011) | <https://www.ncbi.nlm.nih.gov/pmc/articles/PMC3283097/> | <https://www.ncbi.nlm.nih.gov/pmc/articles/PMC7285403/> |
|  | Birth Trauma (% of women who gave birth) | Incidence | 4.4% | 4.4% |  | 2018 | 4.4% |  | (Valdes et al., 2018) | <https://pubmed.ncbi.nlm.nih.gov/29742294/> |  |
|  | Diarrhoeal diseases (general population) | Prevalence | 12.0% | 4.0% | 12.0% | 2014 | 4.0% | 2012 | (GSS et al., 2015) | <https://www.researchgate.net/publication/313517537_Diarrhoea_morbidity_patterns_in_Central_Region_of_Ghana> |  |
|  | Diabetes Mellitus | Prevalence | 6.5% | 6.3% | 6.5% | 2020 | 6.3% | 1998 | (Asamoah-Boaheng et al., 2019) | <https://academic.oup.com/inthealth/article/11/2/83/5115490> | <https://www.sciencedirect.com/science/article/abs/pii/S0033350607002053> |
|  | Meningitis | Incidence | 0.214% | 0.006% | 0.214% | 2016 |  |  | (Domo et al., 2017; Nuoh et al., 2016) | <https://www.ncbi.nlm.nih.gov/pmc/articles/PMC5292117/> | <https://www.ncbi.nlm.nih.gov/pmc/articles/PMC5870784/> |
|  | Endocrine Disorders | Prevalence | 13.1% | 2.2% | 13.1% | 2017 |  |  | (Sarfo-Kantanka et al., 2017) | <https://www.ncbi.nlm.nih.gov/pmc/articles/PMC5343284/> |  |
|  | Kidney Disease (% diabetics and hypertensives) | Prevalence | 13.3% | 13.3% | 30.0% | 2018 | 13.3% | 2015 | (Adjei et al., 2018; Ephraim et al., 2015) | <https://www.ncbi.nlm.nih.gov/pmc/articles/PMC4630826/> | <https://academic.oup.com/ndt/article/33/10/1812/4803283> |
|  | Violence | Incidence | 17.0% | 40.0% | 33.6% | 2016 | 40.0% | 2008 | (GSS et al., 2009; Owusu Adjah and Agbemafle, 2016) | <https://bmcpublichealth.biomedcentral.com/articles/10.1186/s12889-016-3041-x> |  |
|  | Alzheimers & Dementia | Prevalence | 19.2% | 13.4% | 19.2% | 2020 | 13.4% | 2010 | (Cleret de Langavant et al., 2020) | <https://www.ncbi.nlm.nih.gov/pmc/articles/PMC7453145/> |  |
|  | Fall-related injuries | Incidence | 2.6% |  |  | 2015 |  |  | (Stewart Williams et al., 2015) | <https://bmcmedicine.biomedcentral.com/articles/10.1186/s12916-015-0390-8> |  |
|  | Maternal Conditions | Prevalence | 12.0% | 12.0% | 12.0% | 2017 | 12.0% |  | (GSS et al., 2018: 117) | <https://dhsprogram.com/publications/publication-fr340-other-final-reports.cfm> |  |
|  | Hypertension | Prevalence | 30.0% | 19% | 55% | 2019 |  |  | (Bosu et al., 2019; Sanuade et al., 2018) | <https://www.ncbi.nlm.nih.gov/pmc/articles/PMC3645150/?report=reader> | <https://journals.plos.org/plosone/article?id=10.1371/journal.pone.0205985> |
|  | Inflammatory Heart diseases | Prevalence | 6.1% |  |  | 2005 |  |  | (Akosa and Armah, 2005) | <https://www.ncbi.nlm.nih.gov/pmc/articles/PMC1790827/> |  |
|  | Cervical Cancer (15 – 49 years) | Incidence | 0.0264% |  |  | 2011 |  |  | (MOH, 2011) |  |  |
|  | Asthma (general population) | Prevalence | 2.2% |  |  | 2020 |  |  | (Aheto et al., 2020) | <https://www.researchgate.net/publication/236688411_A_Review_of_Epidemiological_Studies_of_Asthma_in_Ghana> |  |
|  | Breast Cancer | Prevalence | 19.7% | 19.7% | 33.9% | 2019 |  |  | (Laryea et al., 2014) | <https://bmccancer.biomedcentral.com/articles/10.1186/s12885-019-5480-0> | <https://bmccancer.biomedcentral.com/articles/10.1186/1471-2407-14-362> |
|  | Peptic Ulcer Disease (15+ years) | Prevalence | 9.0% | 9.0% | 24.5% | 2019 |  |  | (Archampong et al., 2016, 2019) | <https://www.ncbi.nlm.nih.gov/pmc/articles/PMC4994551/> | <https://www.ncbi.nlm.nih.gov/pmc/articles/PMC6639557/> |
|  | Skin Disease (general population) | Prevalence | 5.1% | 2.3% | 5.1% | 2019 |  |  | (Ampah et al., 2016; Kaburi et al., 2019; Rosenbaum et al., 2017) | <https://www.ncbi.nlm.nih.gov/pmc/articles/PMC6567626/> | <https://www.ncbi.nlm.nih.gov/pmc/articles/PMC4831816/> |
|  | Schistosomiasis (school children, 7 -18 years) | Prevalence | 10.3% | 7.8% | 13.3% | 2019 |  |  | (Abaka-Yawson et al., 2019) | <https://www.journalajmah.com/index.php/AJMAH/article/view/30128> |  |
|  | Prostate Cancer | Incidence per 100,000 | 8.8 |  |  | 2013 |  |  | (O’Brien et al., 2013) | <https://www.ncbi.nlm.nih.gov/pmc/articles/PMC4274943/> | <https://bmccancer.biomedcentral.com/articles/10.1186/1471-2407-14-362> |
|  | Hepatitis B (general population) | Prevalence | 12.3% | 7.2% | 14.3% | 2019 | 7.2% | 2015 | (Abesig et al., 2020; Ofori-Asenso and Agyeman, 2016) | <https://journals.plos.org/plosone/article?id=10.1371%2Fjournal.pone.0234348> |  |
|  | Drug Use (Adolescents and adults) | Prevalence | 5.2% | 5.2% | 16.2% | 2014 | 5.2% | 2012 | (Doku et al., 2012; Oppong Asante et al., 2014) | <https://www.afro.who.int/health-topics/substance-abuse> | <https://www.ncbi.nlm.nih.gov/pmc/articles/PMC4258041/> |
|  | Colon-Rectum Cancers (adults) | Prevalence | 0.01% |  |  | 2010 |  |  | (Dakubo et al., 2010) | <http://ugspace.ug.edu.gh/handle/123456789/3635> | <https://pubmed.ncbi.nlm.nih.gov/20665462/> |
|  | Epilepsy (general population) | Prevalence | 1.00% |  |  | 2015 |  |  | (Ae-Ngibise et al., 2015) | <https://www.afro.who.int/publications/fight-against-epilepsy-initiative-ghana> | <https://www.ncbi.nlm.nih.gov/pmc/articles/PMC4561141/> |
|  | Rheumatic Heart Disease (adults) | Prevalence | 3.60% |  |  | 2005 |  |  | (Akosa and Armah, 2005) | <https://www.ncbi.nlm.nih.gov/pmc/articles/PMC1790827/> |  |
|  | Stomach Cancer (adults) | Prevalence | 5.3% |  |  |  |  | 2012 | (Laryea et al., 2014) | <https://bmccancer.biomedcentral.com/articles/10.1186/1471-2407-14-362> |  |
|  | Oral Cancer | Prevalence | 7.4% | 3.5% | 7.4% | 2020 | 3.5% | 2015 | (Owusu-Afriyie et al., 2020) | <https://bmcresnotes.biomedcentral.com/articles/10.1186/s13104-020-05233-9> |  |
|  | Other Neoplasms | Prevalence | 1.4% |  |  | 2015 |  |  | (Stefan, 2015) | <https://academic.oup.com/tropej/article/61/3/165/1714119> |  |
|  | Lung Cancers | Prevalence | 5.3% |  |  |  |  | 2012 | (Laryea et al., 2014) | <https://bmccancer.biomedcentral.com/articles/10.1186/1471-2407-14-362> |  |
|  | Alcohol use (15+ years) | Prevalence | 2.7% | 5.3% | 2.7% | 2016 | 5.3% | 2010 | (World Health Organization et al., 2018) | https://www.who.int/publications/i/item/9789241565639 |  |
|  | Ovary Cancer | Prevalence | 11.3% |  |  | 2012 |  |  | (Laryea et al., 2014) | <https://bmccancer.biomedcentral.com/articles/10.1186/1471-2407-14-362> |  |
|  | Sickle Cell Disease | Prevalence | 2.0% |  |  | 2013 |  |  | (Ohene-Frempong et al., 2008; Piel et al., 2013) | [https://www.ncbi.nlm.nih.gov/pmc/articles/PMC3282939/#:~:text=Sickle%20Cell%20Disease%20%28SCD%29%20is%20a%20significant%20public,Ghanaians%20has%20the%20hemoglobin%20S%20and%2For%20C%20gene.](https://www.ncbi.nlm.nih.gov/pmc/articles/PMC3282939/#:~:text=Sickle%20Cell%20Disease%20%28SCD%29%20is%20a%20significant%20public,Ghanaians%20has%20the%20hemoglobin%20S%20and%2For%20C%20gene. ) |  |
|  | Full vaccination coverage (Penta 3 as proxy) | Coverage | 93.3% |  |  | 2016 | 84.2% | 2006 | (GHS, 2017) |  |  |
|  | Schizophrenia (General population) | Prevalence | 1% | 0.96% | 1% | 2005 | 0.96% | 1984 | (Addo et al., 2013; Read and Doku, 2012; Saha et al., 2005) |  |  |
|  | Depression | Prevalence | 11% |  |  | 2010 |  |  | (Anand, 2015) |  |  |
|  | Children with Stunting | Prevalence | 19% | 19% | 28% | 2014 | 28% | 2008 | (GSS et al., 2009, 2015) |  |  |
|  | Children with wasting | Prevalence | 5% | 5% | 9% | 2014 | 9% | 2008 | (GSS et al., 2009, 2015) |  |  |
|  | Children with Underweight | Prevalence | 11% | 11% | 14% | 2014 | 14% | 2008 | (GSS et al., 2009, 2015) |  |  |
|  | Children with overweight | Prevalence | 3% | 3% | 5% | 2014 | 5% | 2008 | (GSS et al., 2009, 2015) |  |  |
|  | Family planning use (% 15-49 years) | Prevalence (Coverage) | 25.0% | 25.0% | 25.0% | 2017 | 22.0% | 2014 | (GSS et al., 2008, 2015, 2018) |  |  |
|  | Abortion (all forms) - (% of all pregnancies) | Incidence | 22.0% | 22.0% | 22.0% | 2017 | 18.0% | 2007 | (GSS et al., 2008, 2018) | <https://dhsprogram.com/publications/publication-fr340-other-final-reports.cfm> |  |
|  | Miscarriage (% of all pregnancies) | Incidence | 12.0% | 12.0% | 12.0% | 2017 | 9.0% | 2007 | (GSS et al., 2008, 2018) | <https://dhsprogram.com/publications/publication-fr340-other-final-reports.cfm> |  |
|  | Induced abortion (% of all pregnancies) | Incidence | 10.0% | 10.0% | 10.0% | 2017 | 7.0% |  | (GSS et al., 2008, 2018) | <https://dhsprogram.com/publications/publication-fr340-other-final-reports.cfm> |  |
|  | Still births (% of all pregnancies) | Incidence | 2.0% | 2.0% | 2.0% | 2017 | 2.0% |  | (GSS et al., 2008, 2018) | <https://dhsprogram.com/publications/publication-fr340-other-final-reports.cfm> |  |
|  | Antenatal care (at least 4 visits) | Coverage | 89.0% | 89.0% | 89.0% | 2017 | 77.0% | 2007 | (GSS et al., 2008, 2018) | <https://dhsprogram.com/publications/publication-fr340-other-final-reports.cfm> |  |
|  | Skilled Birth Attendance (% of all pregnancies) | Coverage | 79.0% | 79.0% | 79.0% | 2017 | 55.0% | 2007 | (GSS et al., 2008, 2018) | <https://dhsprogram.com/publications/publication-fr340-other-final-reports.cfm> |  |
|  | Spontaneous vaginal delivery (% of all pregnancies) | Incidence | 80.0% | 80.0% | 80.0% | 2017 | 85.0% | 2007 | (GSS et al., 2008, 2018) | <https://dhsprogram.com/publications/publication-fr340-other-final-reports.cfm> |  |
|  | Assisted deliveries (instrumental) (% of all pregnancies) | Incidence | 4.0% | 4.0% | 4.0% | 2017 | 3.0% | 2007 | (GSS et al., 2008, 2018) | <https://dhsprogram.com/publications/publication-fr340-other-final-reports.cfm> |  |
|  | Indications for caesarian section (% of all deliveries) | Incidence | 16.0% | 16.0% | 16.0% | 2017 | 12.0% | 2007 | (GSS et al., 2008, 2018) | <https://dhsprogram.com/publications/publication-fr340-other-final-reports.cfm> |  |
|  | Complications of pregnancy (% of all pregnancies) | Prevalence | 19.0% | 19.0% | 19.0% | 2017 | 17.0% | 2007 | (GSS et al., 2008, 2018) | <https://dhsprogram.com/publications/publication-fr340-other-final-reports.cfm> |  |
|  | Hemorrhage (mostly bleeding after childbirth) - (% of all pregnancies) | Incidence | 5.7% | 5.7% | 5.7% | 2017 | 5.0% | 2007 | (GSS et al., 2008, 2018) | <https://dhsprogram.com/publications/publication-fr340-other-final-reports.cfm> |  |
|  | Puerperal sepsis (infections usually after childbirth) - (% of all pregnancies) | Prevalence | 3.2% | 2.0% | 5.0% | 2015 | 5.0% | 2007 | (GSS et al., 2008, 2015, 2018) | <https://dhsprogram.com/publications/publication-fr340-other-final-reports.cfm> |  |
|  | Oedema and high blood pressure during pregnancy (pre-eclampsia) - (% of all pregnancies) | Prevalence | 2.5% | 2.5% | 2.5% | 2017 | 2.0% | 2007 | (GSS et al., 2008, 2018) | <https://dhsprogram.com/publications/publication-fr340-other-final-reports.cfm> |  |
|  | Eclampsia during delivery (% of all deliveries) | Prevalence | 1.9% | 1.9% | 1.9% | 2017 | 1.0% | 2007 | (GSS et al., 2008, 2018) | <https://dhsprogram.com/publications/publication-fr340-other-final-reports.cfm> |  |
|  | Other complications of pregnancy and delivery (% of all deliveries) | Prevalence | 4.2% | 4.2% | 4.2% | 2017 | 9.0% | 2007 | (GSS et al., 2008, 2018) | <https://dhsprogram.com/publications/publication-fr340-other-final-reports.cfm> |  |
|  | Postnatal care of the Newborn (% of all live births) | Coverage | 84.0% | 84.0% | 84.0% | 2017 | 60.0% | 2007 | (GSS et al., 2008, 2018) | <https://dhsprogram.com/publications/publication-fr340-other-final-reports.cfm> |  |
|  | Ectopic pregnancies (% of all pregnancies) | Prevalence | 4.0% |  |  | 2015 |  |  | (GSS et al., 2015) | <https://dhsprogram.com/publications/publication-fr340-other-final-reports.cfm> |  |
|  | Acute Ear Infection | Prevalence | 0.88% |  |  |  |  |  |  | <https://bmcinfectdis.biomedcentral.com/articles/10.1186/s12879-020-4950-y> |  |
|  | Acute Urinary Tract Infection | Prevalence | 15.9% |  |  | 2015 |  |  | (Donkor et al., 2019) | <https://www.ncbi.nlm.nih.gov/pmc/articles/PMC6628945/> |  |
|  | Anaemia in pregnancy | Prevalence | 33.0% |  |  | 2019 |  |  |  | <https://pubmed.ncbi.nlm.nih.gov/32153953/> |  |
|  | Intestinal Worms | Prevalence | 14.3% |  |  | 2020 |  |  | (Abaka-Yawson et al., 2020) | <https://www.hindawi.com/journals/jeph/2020/9315025/> |  |
|  | Malaria in Pregnancy | Prevalence | 2.0% |  |  | 2019 |  |  |  | <https://pubmed.ncbi.nlm.nih.gov/32153953/> |  |
|  | Pneumonia | Prevalence | 18.4% |  |  | 2018 |  |  | (Osei et al., 2018) | <https://myjournal.afrijcmr.org/index.php/ajcmr/article/view/28> |  |
|  | Rheumatic and Joint Diseases (50 years +) | Prevalence | 16.56% |  |  | 2007 |  |  | (Brennan-Olsen et al., 2017) | <https://bmcmusculoskeletdisord.biomedcentral.com/articles/10.1186/s12891-017-1624-z> |  |
|  | Septicaemia | Prevalence | 18.4% | 10.5% | 35.2% | 2016 |  |  | (Lester et al., 2020) | <https://academic.oup.com/jac/article/75/3/492/5632029> |  |
|  | Typhoid Fever | Prevalence | 2.1% | 0.1% | 2.1% | 2016 | 0.1% | 2012 | (Fusheini and Gyawu, 2020) | <https://www.annalsofglobalhealth.org/articles/10.5334/aogh.2833/> |  |
|  | Upper respiratory tract infection | Prevalence | 22% | 16.2% | 47.3% | 2014 | 12.3% | 1993 | (Seidu et al., 2019) | <https://www.ncbi.nlm.nih.gov/pmc/articles/PMC6558297/> |  |
|  | Vaginal Discharge | Prevalence | 56.4% | 56.4% | 66% | 2019 |  |  | (Aubyn and Tagoe, 2013; Konadu et al., 2019) | <https://bmcpregnancychildbirth.biomedcentral.com/articles/10.1186/s12884-019-2488-z> | <https://www.ncbi.nlm.nih.gov/pmc/articles/PMC4027303/> |
|  | General Anaemia | Prevalence | 42.4% | 41.2% | 43.9% | 2014 | 58.7% | 2008 | GSS et al., 2009, 2015) |  |  |
|  | Overweight and obesity | Prevalence | 30% | 19% | 55% | 2016 |  |  | (Ofori-Asenso et al., 2016) |  |  |
|  | Hernia | Prevalence | 10.8% | 8% | 13.6% | 2016 |  |  | (Ohene-Yeboah et al., 2016) | <https://pubmed.ncbi.nlm.nih.gov/26578320/> |  |

**REFERENCES**

Abaka-Yawson A, Hotorvi C, Oduro EA, et al. (2019) Prevalence and Associated Factors of Urinary Schistosomiasis among Basic School Children in the Akyemansa District, Ghana. *Asian Journal of Medicine and Health*: 1–10. DOI: 10.9734/ajmah/2019/v15i430128.

Abaka-Yawson A, Sosu SQ, Kwadzokpui PK, et al. (2020) Prevalence and Determinants of Intestinal Parasitic Infections among Pregnant Women Receiving Antenatal Care in Kasoa Polyclinic, Ghana. Hindawi. DOI: https://doi.org/10.1155/2020/9315025.

Abesig J, Chen Y, Wang H, et al. (2020) Prevalence of viral hepatitis B in Ghana between 2015 and 2019: A systematic review and meta-analysis. *PLOS ONE* 15(6). Public Library of Science: e0234348. DOI: 10.1371/journal.pone.0234348.

Abubakari A, Kynast-Wolf G and Jahn A (2015) Prevalence of abnormal birth weight and related factors in Northern region, Ghana. *BMC Pregnancy and Childbirth* 15. DOI: 10.1186/s12884-015-0790-y.

Addo KK, Addo SO, Bonsu C, et al. (2019) Population-Based Tuberculosis Disease Prevalence Survey in Ghana: The Role and Lessons Learnt from the Laboratory. *Journal of Tuberculosis Research* 7(2). 2. Scientific Research Publishing: 95–108. DOI: 10.4236/jtr.2019.72009.

Addo R, Nonvignon J and Aikins M (2013) Household costs of mental health care in Ghana. *J Ment Health Policy Econ* 16(4): 151–9.

Adjei DN, Stronks K, Adu D, et al. (2018) Chronic kidney disease burden among African migrants in three European countries and in urban and rural Ghana: the RODAM cross-sectional study. *Nephrology Dialysis Transplantation* 33(10): 1812–1822. DOI: 10.1093/ndt/gfx347.

Ae-Ngibise KA, Akpalu B, Ngugi A, et al. (2015) Prevalence and risk factors for Active Convulsive Epilepsy in Kintampo, Ghana. *The Pan African Medical Journal* 21. DOI: 10.11604/pamj.2015.21.29.6084.

Aheto JMK, Udofia EA, Kallson E, et al. (2020) Prevalence, socio-demographic and environmental determinants of asthma in 4621 Ghanaian adults: Evidence from Wave 2 of the World Health Organization’s study on global AGEing and adult health. *PLOS ONE* 15(12). Public Library of Science: e0243642. DOI: 10.1371/journal.pone.0243642.

Akosa A and Armah H (2005) Cardiomegaly in Ghana: An Autopsy Study. *Ghana Medical Journal* 39(4): 122–127. Available at: https://www.ncbi.nlm.nih.gov/pmc/articles/PMC1790827/ (accessed 3 January 2021).

Ampah KA, Asare P, Binnah DD-G, et al. (2016) Burden and Historical Trend of Buruli Ulcer Prevalence in Selected Communities along the Offin River of Ghana. *PLoS Neglected Tropical Diseases* 10(4). DOI: 10.1371/journal.pntd.0004603.

Anand A (2015) Understanding Depression among Older Adults in Six Low-Middle Income Countries using WHO-SAGE Survey. *Behavioral Health* 1.

Appiah LT, Sarfo FS, Agyemang C, et al. (2017) Current trends in admissions and outcomes of cardiac diseases in Ghana. *Clinical Cardiology* 40(10): 783–788. DOI: https://doi.org/10.1002/clc.22753.

Archampong TN, Asmah RH, Richards CJ, et al. (2019) Gastro-duodenal disease in Africa: Literature review and clinical data from Accra, Ghana. *World Journal of Gastroenterology* 25(26): 3344–3358. DOI: 10.3748/wjg.v25.i26.3344.

Archampong TNA, Asmah RH, Wiredu EK, et al. (2016) Factors associated with gastro-duodenal disease in patients undergoing upper GI endoscopy at the Korle-Bu Teaching Hospital, Accra, Ghana. *African Health Sciences* 16(2): 611–619. DOI: 10.4314/ahs.v16i2.32.

Asamoah-Boaheng M, Sarfo-Kantanka O, Tuffour AB, et al. (2019) Prevalence and risk factors for diabetes mellitus among adults in Ghana: a systematic review and meta-analysis. *International Health* 11(2): 83–92. DOI: 10.1093/inthealth/ihy067.

Aubyn GB and Tagoe DNA (2013) Prevalence of vaginal infections and associated lifestyles of students in the university of Cape Coast, Ghana. *Asian Pacific Journal of Tropical Disease* 3(4): 267–270. DOI: 10.1016/S2222-1808(13)60068-7.

Bosu WK, Reilly ST, Aheto JMK, et al. (2019) Hypertension in older adults in Africa: A systematic review and meta-analysis. *PloS one* 14(4). Public Library of Science.

Brennan-Olsen SL, Cook S, Leech MT, et al. (2017) Prevalence of arthritis according to age, sex and socioeconomic status in six low and middle income countries: analysis of data from the World Health Organization study on global AGEing and adult health (SAGE) Wave 1. *BMC Musculoskeletal Disorders* 18(1): 271. DOI: 10.1186/s12891-017-1624-z.

Cleret de Langavant L, Bayen E, Bachoud‐Lévi A, et al. (2020) Approximating dementia prevalence in population‐based surveys of aging worldwide: An unsupervised machine learning approach. *Alzheimer’s & Dementia : Translational Research & Clinical Interventions* 6(1). DOI: 10.1002/trc2.12074.

Dakubo JC, Naaeder SB, Tettey Y, et al. (2010) Colorectal carcinoma: an update of current trends in Accra. *West African Journal of Medicine* 29(3): 178–183. DOI: 10.4314/wajm.v29i3.68218.

Doku D, Koivusilta L and Rimpelä A (2012) Socioeconomic differences in alcohol and drug use among Ghanaian adolescents. *Addictive Behaviors* 37(3): 357–360. DOI: 10.1016/j.addbeh.2011.11.020.

Domo NR, Nuolabong C, Nyarko KM, et al. (2017) Uncommon mixed outbreak of pneumococcal and meningococcal meningitis in Jirapa District, Upper West Region, Ghana, 2016. *Ghana Medical Journal* 51(4): 149–155. Available at: https://www.ncbi.nlm.nih.gov/pmc/articles/PMC5870784/ (accessed 3 January 2021).

Donkor ES, Horlortu PZ, Dayie NT, et al. (2019) Community acquired urinary tract infections among adults in Accra, Ghana. *Infection and Drug Resistance* 12: 2059–2067. DOI: 10.2147/IDR.S204880.

Ephraim RK, Biekpe S, Sakyi SA, et al. (2015) Prevalence of chronic kidney disease among the high risk population in South-Western Ghana; a cross sectional study. *Canadian Journal of Kidney Health and Disease* 2. DOI: 10.1186/s40697-015-0076-3.

Fusheini A and Gyawu SK (2020) Prevalence of Typhoid and Paratyphoid Fever in the Hohoe Municipality of the Volta Region, Ghana: A Five-Year Retrospective Trend Analysis. *Annals of Global Health* 86(1). 1. Ubiquity Press: 111. DOI: 10.5334/aogh.2833.

Ghana AIDS Commission (2019) 2019 National HIV Estimates and Projection. Accra. Available at: https://www.ghanaids.gov.gh/pages/fact-sheets-reports (accessed 3 January 2021).

GHS (2017) *Facts and Figures*. Accra: Ghana Health Service.

GSS, GHS, and ICF International (2009) *Ghana Demographic and Health Survey 2008.* Survey. Rockville, Maryland, USA: Ghana Statistical Service. Available at: https://dhsprogram.com/publications/publication-FR307-DHS-Final-Reports.cfm.

GSS, GHS, and ICF International (2015) *Ghana Demographic and Health Survey 2014.* Survey. Rockville, Maryland, USA: Ghana Statistical Service. Available at: https://dhsprogram.com/publications/publication-FR307-DHS-Final-Reports.cfm.

GSS GSS-, GHS GHS- and ICF (2008) Ghana Maternal Health Survey 2007. Available at: https://www.dhsprogram.com/publications/publication-fr340-other-final-reports.cfm (accessed 12 June 2020).

GSS GSS-, GHS GHS- and ICF (2018) Ghana Maternal Health Survey 2017. Available at: https://www.dhsprogram.com/publications/publication-fr340-other-final-reports.cfm (accessed 12 June 2020).

Kaburi BB, Ameme DK, Adu-Asumah G, et al. (2019) Outbreak of scabies among preschool children, Accra, Ghana, 2017. *BMC Public Health* 19. DOI: 10.1186/s12889-019-7085-6.

Konadu DG, Owusu-Ofori A, Yidana Z, et al. (2019) Prevalence of vulvovaginal candidiasis, bacterial vaginosis and trichomoniasis in pregnant women attending antenatal clinic in the middle belt of Ghana. *BMC Pregnancy and Childbirth* 19(1): 341. DOI: 10.1186/s12884-019-2488-z.

Konlan Kennedy Diema, Doat AR, Mohammed I, et al. (2020) Prevalence and Pattern of Road Traffic Accidents among Commercial Motorcyclists in the Central Tongu District, Ghana. *The Scientific World Journal* 2020. DOI: 10.1155/2020/9493718.

Kudebong M, Wurapa F, Nonvignon J, et al. (2011) Economic Burden of Motorcycle Accidents in Northern Ghana. *Ghana Medical Journal* 45(4): 135–142. Available at: https://www.ncbi.nlm.nih.gov/pmc/articles/PMC3283097/ (accessed 3 January 2021).

Laryea DO, Awuah B, Amoako YA, et al. (2014) Cancer incidence in Ghana, 2012: evidence from a population-based cancer registry. *BMC Cancer* 14(1): 362. DOI: 10.1186/1471-2407-14-362.

Lester R, Musicha P, van Ginneken N, et al. (2020) Prevalence and outcome of bloodstream infections due to third-generation cephalosporin-resistant Enterobacteriaceae in sub-Saharan Africa: a systematic review. *Journal of Antimicrobial Chemotherapy* 75(3): 492–507. DOI: 10.1093/jac/dkz464.

MOH (2011) *National Strategy for Cancer Control in Ghana, 2012 - 2016*. Accra: Ministry of Health, Ghana.

Nuoh RD, Nyarko KM, Nortey P, et al. (2016) Review of meningitis surveillance data, upper West Region, Ghana 2009-2013. *The Pan African Medical Journal* 25(Suppl 1). DOI: 10.11604/pamj.supp.2016.25.1.6180.

O’Brien KS, Soliman AS, Awuah B, et al. (2013) Establishing Effective Registration Systems in Resource-Limited Settings: Cancer Registration in Kumasi, Ghana. *Journal of registry management* 40(2): 70–77. Available at: https://www.ncbi.nlm.nih.gov/pmc/articles/PMC4274943/ (accessed 3 January 2021).

Ofori-Asenso R and Agyeman AA (2016) Hepatitis B in Ghana: a systematic review & meta-analysis of prevalence studies (1995-2015). *BMC Infectious Diseases* 16(1): 130. DOI: 10.1186/s12879-016-1467-5.

Ofori-Asenso R, Agyeman AA, Laar A, et al. (2016) Overweight and obesity epidemic in Ghana—a systematic review and meta-analysis. *BMC Public Health* 16(1): 1239. DOI: 10.1186/s12889-016-3901-4.

Ohene-Frempong K, Oduro J, Tetteh H, et al. (2008) SCREENING NEWBORNS FOR SICKLE CELL DISEASE IN GHANA. *Pediatrics* 121(Supplement 2). Am Acad Pediatrics: S120–S121.

Ohene-Yeboah M, Beard JH, Frimpong-Twumasi B, et al. (2016) Prevalence of Inguinal Hernia in Adult Men in the Ashanti Region of Ghana. *World Journal of Surgery* 40(4): 806–812. DOI: 10.1007/s00268-015-3335-7.

Oppong Asante K, Meyer-Weitz A and Petersen I (2014) Substance use and risky sexual behaviours among street connected children and youth in Accra, Ghana. *Substance Abuse Treatment, Prevention, and Policy* 9. DOI: 10.1186/1747-597X-9-45.

Osei FA, Mensah KA, Ansong D, et al. (2018) Prevalence of pneumonia and risk factors of pneumonia mortality among children under five years. *African Journal of Current Medical Research* 2(2). 2. DOI: 10.31191/afrijcmr.v2i2.28.

Owusu Adjah ES and Agbemafle I (2016) Determinants of domestic violence against women in Ghana. *BMC Public Health* 16(1): 368. DOI: 10.1186/s12889-016-3041-x.

Owusu-Afriyie O, Owiredu WKBA, Oti AA, et al. (2020) Survival rates of head and neck cancers in Ghana: a retrospective study at the Komfo Anokye Teaching Hospital. *BMC Research Notes* 13(1): 392. DOI: 10.1186/s13104-020-05233-9.

Piel FB, Patil AP, Howes RE, et al. (2013) Global epidemiology of sickle haemoglobin in neonates: a contemporary geostatistical model-based map and population estimates. *The Lancet* 381(9861): 142–151. DOI: 10.1016/S0140-6736(12)61229-X.

Read UM and Doku VCK (2012) Mental health research in Ghana: A literature review. *Ghana Medical Journal* 46(2). 2: 29–38. DOI: 10.4314/gmj.v46i2.

Rosenbaum BE, Klein R, Hagan PG, et al. (2017) Dermatology in Ghana: a retrospective review of skin disease at the Korle Bu Teaching Hospital Dermatology Clinic. *The Pan African Medical Journal* 26. DOI: 10.11604/pamj.2017.26.125.10954.

Saha S, Chant D, Welham J, et al. (2005) A Systematic Review of the Prevalence of Schizophrenia. *PLOS Medicine* 2(5). Public Library of Science: e141. DOI: 10.1371/journal.pmed.0020141.

Sanuade OA, Boatemaa S and Kushitor MK (2018) Hypertension prevalence, awareness, treatment and control in Ghanaian population: Evidence from the Ghana demographic and health survey. *PLOS ONE* 13(11). Public Library of Science: e0205985. DOI: 10.1371/journal.pone.0205985.

Sanuade OA, Dodoo FN-A, Koram K, et al. (2019) Prevalence and correlates of stroke among older adults in Ghana: Evidence from the Study on Global AGEing and adult health (SAGE). *PLoS ONE* 14(3). DOI: 10.1371/journal.pone.0212623.

Sarfo-Kantanka O, Sarfo FS, Ansah EO, et al. (2017) Spectrum of Endocrine Disorders in Central Ghana. *International Journal of Endocrinology* 2017. DOI: 10.1155/2017/5470731.

Seidu A-A, Ameyaw EK, Ahinkorah BO, et al. (2019) Ecological zone and symptoms of acute respiratory infection among children under five in Ghana: 1993–2014. *SSM - Population Health* 8. DOI: 10.1016/j.ssmph.2019.100414.

Stefan DC (2015) Patterns of Distribution of Childhood Cancer in Africa. *Journal of Tropical Pediatrics* 61(3): 165–173. DOI: 10.1093/tropej/fmv005.

Stewart Williams J, Kowal P, Hestekin H, et al. (2015) Prevalence, risk factors and disability associated with fall-related injury in older adults in low- and middle-incomecountries: results from the WHO Study on global AGEing and adult health (SAGE). *BMC Medicine* 13(1): 147. DOI: 10.1186/s12916-015-0390-8.

Valdes V, Adongo PB, Nwameme AU, et al. (2018) Risk factors for self-reported postpartum hemorrhage in Ga East, Ghana. *International Journal of Gynaecology and Obstetrics: The Official Organ of the International Federation of Gynaecology and Obstetrics* 142(2): 201–206. DOI: 10.1002/ijgo.12523.

World Health Organization, Management of Substance Abuse Team, and World Health Organization (2018) *Global Status Report on Alcohol and Health 2018.* Available at: http://www.who.int/substance_abuse/publications/global_alcohol_report/en/ (accessed 3 January 2021).
