# Supplementary material for: Modelling the supply and need for health professionals for primary health care in Ghana: Implications for health professions education and employment planning
Source: PLoS One. 2021 Sep 28;16(9):e0257957. doi: 10.1371/journal.pone.0257957 (PMC8478216; doi:10.1371/journal.pone.0257957)
Supplement: Attachment — Submitted filename: Response to Review commnents_PONE-D-21-05604_14 August 2021.docx [file pone.0257957.s004.docx]

PONE-D-21-05604

**Modelling the supply and need for health professionals for primary health care in Ghana: Implications for health professions education and employment planning**

Dr Christmal Dela Christmals

PLOS ONE

# **Response to Reviewer comments/remarks**

| **Area** | **Comments/remarks** | **Authors’ Response** |
| --- | --- | --- |
| **General remarks:** | Thank you for the opportunity to review this manuscript.  Health human resource planning and the development of human resource capital remain important. This was again highlighted by the strain the COVID-19 pandemic caused health care facilities.  The manuscript reports on the modelling of the health workforce requirements of the primary health care system in Ghana for the period 2020 to 2035 using a Microsoft Excel-based tool that was developed by the authors. This population needs-based simulation model was recently published in the International Journal of Environmental Research and Public Health. Health professions education is one of the foci of this manuscript. | We thank the reviewer for the thorough review and positive feedback. We are encouraged and grateful. |
|  | Input data for the model appears to be comprehensive and was accumulated from multiple sources. Herein lies the risk: to ensure that input data is current and reliable. | We thank the reviewers and editor for this important advice. As far as possible, we have ensured that we used only the best available, current and reliable data from Ghana in a transparent manner, providing the sources of all input data.  In the spirit of using the most current data, we have cross-checked and made a few and minor updates based on new data from the Ghana Health Service (GHS), which became available during the period the manuscript was undergoing peer-review (we have notified the editorial office of PLOS ONE in an email on 24 June 2021 of this update). We have, therefore, slightly updated the disease burden component of the excel model and incorporated the resulting updates in the model output in the revised manuscript. To underscore the value of periodic updates of the analysis, we have included a recommendation to that effect, especially after every major national health survey, when significant technological or clinical practice changes are adopted, when scope of practice for health professionals evolve, when significant curriculum changes are made, and when important population health events occur, such as epidemics, disasters etc. (see lines 1281 – 1309 under conclusion). |
| **Language:** | Sentences were frequently long and somewhat challenging to comprehend on the first read. | We have revised the manuscript to address the issues of long sentences, typographical and grammatical errors. It is our sincere hope that the manuscript is now much clearer and reader-friendly. |
|  | There were typographical or grammatical errors in the manuscript that should be corrected to ensure that the text is clear and easy to read. Examples include lines 76-79, 89, 112-113, 179-183, 260-261, 458-463, 478-480, 547-548, 556-558, 585-588, 630-631… | We thank the reviewer for meticulously pointing out these issues. We have revised these sentences and corrected the typographical errors accordingly. |
|  | Abbreviations should be written in full the first time they are used in the text (e.g. UHC, WISN, STEPS, SAR) and should be used consistently (e.g. MoH vs. MOH). | We have defined all the abbreviations at first mention and also ensured consistency in the use of ‘MOH’ throughout the manuscript. |
|  | Recommend that the word folds be amended to fold throughout the manuscript e.g. lines 53, 87, 289, 307, 308, 326, 338. | We are grateful to the reviewer for the guidance. We have accordingly amended. |
|  | Other language changes recommended:   - Line 101: The questions include, amongst others,… - Line 104: remove the full stop after the question mark - Line 111: requires a conjunction like the word **and** - Line 260: spelling of the word **subsidizes** - Line 289: spelling of the word **baseline** | We have made the corrections as guided by the esteemed reviewer. Thank you. |
|  | Line 291 and elsewhere: reconsider the use of apostrophes in the text e.g. General Practitioners stock vs. General Practitioners’ stock | We have ensured consistency by eliminating the use of apostrophes. |
|  | - Line 325: …in 2020 and **is** expected to… - Line 396-7: decline ~~by~~ **to** 45% - Line 485: **US**$ - Line 488: eleven health professional **groups** or eleven **groups of** health professionals. - Line 523: improve the overall **~~the~~** supply to need - Line 545: on **~~a~~** utilisation-oriented health facility staffing norms | Thank you for this tremendous support. The errors have been fixed as guided. |
| **Tables and Figures:** | Tables and Figures supported the text well.  Table 1 claims that:   a. The annual cost associated with training of General Practitioners was less than that of Physician Assistants, Midwife and Nurse groups. Is that accurate? b. That the attrition rate of four groups of health care providers (i.e. Physician Assistants, Nutrition and Dieticians, Biomedical Scientists and Laboratory Technicians) was 0.0%. Is that accurate? c. Also, why would the retirement/death rate of the Midwife group far exceed that of other groups at 7.6%? Does the age of this cohort differ statistically from that of the Nurse groups? Do younger health care professionals choose not to do midwifery? | Thank you for these observations.   a. Indeed, it would seem ironical, but we have cross-checked from the sources we obtained the data, and it is correct. As explained in the methodology, these costs were taken as an average from two public universities and one private university. For General Practitioners, the cost of training at the University of Ghana Medical School is highly subsidized and hence slightly influenced the average cost. We have made a footnote to explain it. b. Indeed, for the other forms of attrition (besides deaths and retirements), these cades had 0.0% from the available data. The other forms of attrition included resignations, vacation of posts and dismissals – we have included a footnote to explain this. It would be noted that in the overall attrition rate, we combined the deaths, retirements and other forms of attrition, which none of the groups of health professionals had 0.0%. c. For midwives, their average age is much higher than the rest of the professionals, and this has over the years resulted in a higher attrition rate as compared to the others. See our earlier commentary n this issue: <https://doi.org/10.1186/s12960-019-0377-1> |
|  | The order in which the health professional groups are presented in the Tables is inconsistent across tables. Personally, I (and perhaps other readers) would prefer if it were the same. | As advised, we have re-arranged the categories of health professionals in alphabetical order across all tables. |
|  | List abbreviations in the legend below tables e.g. SAR in Table 4. | We have added the full meaning of SAR in table 4. |
|  | Productivity and workload was not expressed as full time equivalents and may make comparisons complex. Also the activity standards and standard workloads captured in the model allows for variable costing that may significantly alter cost as a function of productivity. | Usually, productivity is measured in terms of the number of units of service outputs per unit time, which in this model, we used the activity standards as the proxy. However, the estimated needs-based requirements are measured in terms of full-time equivalence (FTE). |
|  | One assumes that the quality of health of Ghanaians would improve should the needs-based approach be implemented. This would negate the findings of a 15-year modelling exercise and calls for re-analysis in a more dynamic fashion. Is this part of the intention of the Microsoft Excel-based tool? | Indeed, as the level of health of the population evolves, it would have a different impact on the need for health professionals. For example, reduction in disease burden would reduce the need for curative health professionals but could also increase the need for other care professionals to cater for the aged and those with long-term conditions. These have been taken into account in the model. It is also against this backdrop that we recommend a revision of the analysis at regular intervals. |
| **References:** | The references require a major overall.   - Reports should be referenced as such but with consistency (preferably written in full) e.g. 6. WHO… and 9. World Health Organization. It may also be advantageous to add the URL for these references. - Reference 2 has been duplicated as 33 - Reference 3 has been duplicated as 25 and again as 37! - Reference 5 has been duplicated as 34 - Reference 10 has been duplicated as 17 and again as 59! - Reference 14 has been duplicated as 18 - Reference 26 has been duplicated as 38 - Reference 28 has been duplicated as 36 and again as 39! - Reference 29 has been duplicated as 35 - Reference 12 should be cited as: Saleh, Karima. 2013. The Health Sector in Ghana: A Comprehensive Assessment. Directions in development: human development;. Washington, DC: World Bank. © World Bank. https://openknowledge.worldbank.org/handle/10986/12297 License: CC BY 3.0 IGO. - Reference 30 is incomplete and should be cited as Asamani JA, Christmals CD. Reitsma GM. Advancing the population needs-based health workforce planning methodology: A simulation tool for country application. Int. J. Environ. Res. Public Health. 2021;18(4). | We have addressed all these referencing issues (and others) outlined by the esteemed reviewer. We would like to also highlight that some of the institutional reports from the Government were collected from the shelves of the MOH, GHS and other institutions during the study. Those documents are unavailable on the web and hence have no web links to be included in the reference list. |

**PEER-REVIEWERS ANSWERS TO JOURNAL QUESTIONS**

| **Reviewer** | **Comments/remarks** | **Authors’ Response** |
| --- | --- | --- |
| Reviewer #1 | Congratulations for tackling a difficult but extremely relevant subject. Planning for healthcare needs, if done properly offers all stakeholders an objective tool on which to base key decisions including resource allocation. The human resource is the most important component of such plans | Thank you for the positive feedback. We are encouraged by it. |
|  | The writing style, grammar, spelling and formatting are all exceptionally well done, save for a few minor errors. | We have revised manuscript and corrected the errors that have been identified. |
|  | Figures and illustrations are appropriate and relevant to the points advanced in the text | We appreciate this positive feedback. |
|  | This reviewer did not have the capacity to confirm the veracity and assumptions behind the extensively used mathematical formulae. These formulae are the single most important tool used to arrive at the various conclusions made. However, the notes and explanations were reviewed in-depth and each equation was interrogated. The equations are easy to understand and logical. This aspect of the research therefore stands out as being particularly innovative | We thank the esteemed reviewer for the thorough and meticulous assessment of the mathematical formulae that underpin the excel model. We are greatly encouraged by the positive feedback. |
|  | The discussion on the type of healthcare professional needed, is weakened by the fact that some assumptions made are speculative. For instance, the scope of practice of the Biomedical Scientist vs Laboratory Technician which is based on Ghana Government (Employer) publications may not be factual. This is an important factor to consider in needs analysis. | Thank you for this important observation. We acknowledge that the scope of practice of health professionals such as Biomedical Scientists and Laboratory Technicians is dynamic and evolves with time. However, at the time of the analysis, the Ghana Government (Employer) publications was the main scope of practice guidance for these professionals in the country. We have therefore, taken the reviewer’s comment into account and made a recommendation to update the analysis when clinical practice or scope of practice changes (see lines 1281 – 1309 under conclusion). |
|  | The Limitations are in-depth and mostly factual (partially discussed above). The Conclusion is well structured. Overall, this is an excellent work requiring no specific alteration. | We appreciate this positive feedback. |
| Reviewer #2 | The manuscript appears to be technically sound and the data support the conclusion. Data have been validated and a sensitivity analysis performed. All data underlying the findings of the manuscript are fully available. The statistical analysis is a function of the modelling software used in the study. | We thank the esteemed reviewer for the thorough assessment of the mathematical formulae that underpin the model. We are greatly encouraged by the feedback. |
|  | The ethical aspects of the study have been complied with in that institutional ethic approval has been obtained from both the North West University and Ghana Health service Ethics Committee. Specific comments and recommendations have been highlighted in the attachment. | We appreciate this positive feedback. |
