# Supplementary material for: Modelling the supply and need for health professionals for primary health care in Ghana: Implications for health professions education and employment planning
Source: PLoS One. 2021 Sep 28;16(9):e0257957. doi: 10.1371/journal.pone.0257957 (PMC8478216; doi:10.1371/journal.pone.0257957)
Supplement: Attachment — Submitted filename: PONE-D-21-05604 PLOS ONE Reviewer comments 10082021.docx [file pone.0257957.s003.docx]

PONE-D-21-05604

Modelling the supply and need for health professionals for primary health care in Ghana: Implications for health professions education and employment planning

Dr Christmal Dela Christmals

PLOS ONE

# **Reviewer remarks:**

Thank you for the opportunity to review this manuscript.

Health human resource planning and development of human resource capital remain important. This was again highlighted by the strain the COVID-19 pandemic caused health care facilities.

The manuscript reports on the modelling of the health workforce requirements of the primary health care system in Ghana for the period 2020 to 2035 using a Microsoft Excel-based tool that was developed by the authors. This population needs-based simulation model was recently published in the International Journal of Environmental Research and Public Health. Health professions education is one of the foci of this manuscript.

Input data for the model appears to be comprehensive and was accumulated from multiple sources. Herein lies the risk: to ensure that input data is current and reliable.

# **Specific comments:**

## **Language:**

Sentences were frequently long and somewhat challenging to comprehend on the first read. There were typographical or grammatical errors in the manuscript that should be corrected to ensure that the text is clear and easy to read. Examples include lines 76-79, 89, 112-113, 179-183, 260-261, 458-463, 478-480, 547-548, 556-558, 585-588, 630-631…

Abbreviations should be written in full the first time they are used in the text (e.g. UHC, WISN, STEPS, SAR) and should be used consistently (e.g. MoH vs. MOH).

Recommend that the word folds be amended to fold throughout the manuscript e.g. lines 53, 87, 289, 307, 308, 326, 338.

Other language changes recommended:

Line 101: The questions include, amongst others,…

Line 104: remove full stop after question mark

Line 111: requires a conjunction like the word **and**

Line 260: spelling of the word **subsidizes**

Line 289: spelling of the word **baseline**

Line 291 and elsewhere: reconsider the use of apostrophes in the text e.g. General Practitioners stock vs. General Practitioners’ stock

Line 325: …in 2020 and **is** expected to…

Line 396-7: decline ~~by~~ **to** 45%

Line 485: **US**$

Line 488: eleven health professional **groups** or eleven **groups of** health professionals.

Line 523: improve the overall **~~the~~** supply to need

Line 545: on **~~a~~** utilisation-oriented health facility staffing norms

## **Tables and Figures:**

Tables and Figures supported the text well.

Table 1 claims that:

a. The annual cost associated with training of General Practitioners was less than that of Physician Assistants, Midwife and Nurse groups. Is that accurate?
b. That the attrition rate of four groups of health care providers (i.e. Physician Assistants, Nutrition and Dieticians, Biomedical Scientists and Laboratory Technicians) was 0.0%. Is that accurate? Also, why would the retirement/death rate of the Midwife group far exceed that of other groups at 7.6%? Does the age of this cohort differ statistically from that of the Nurse groups? Do younger health care professionals choose not to do midwifery?

The order in which the health professional groups are presented in the Tables is inconsistent across tables. Personally, I (and perhaps other readers) would prefer if it were the same.

List abbreviations in the legend below tables e.g. SAR in Table 4.

Productivity and workload was not expressed as full time equivalents and may make comparisons complex. Also the activity standards and standard workloads captured in the model allows for variable costing that may significantly alter cost as a function of productivity.

One assumes that the quality of health of Ghanaians would improve should the needs-based approach be implemented. This would negate the findings of a 15-year modelling exercise and calls for re-analysis in a more dynamic fashion. Is this part of the intension of the Microsoft Excel-based tool?

## **References:**

The references require a major overall.

Reports should be referenced as such but with consistency (preferably written in full) e.g. 6. WHO… and 9. World Health Organization. It may also be advantageous to add the URL for these references.

Reference 2 has been duplicated as 33

Reference 3 has been duplicated as 25 and again as 37!

Reference 5 has been duplicated as 34

Reference 10 has been duplicated as 17 and again as 59!

Reference 14 has been duplicated as 18

Reference 26 has been duplicated as 38

Reference 28 has been duplicated as 36 and again as 39!

Reference 29 has been duplicated as 35

Reference 12 should be cited as: Saleh, Karima. 2013. The Health Sector in Ghana: A Comprehensive Assessment. Directions in development: human development;. Washington, DC: World Bank. © World Bank. https://openknowledge.worldbank.org/handle/10986/12297 License: CC BY 3.0 IGO.

Reference 30 is incomplete and should be cited as: Asamani JA, Christmals CD.Reitsma GM. Advancing the population needs-based health workforce planning methodology: A simulation tool for country application. Int. J. Environ. Res. Public Health. 2021;18(4).
